# Inhibitory Mechanisms in the Processing of Negations: A Neural Reuse Hypothesis

**DOI:** 10.1007/s10936-021-09796-x

**Published:** 2021-08-12

**Authors:** David Beltrán, Bo Liu, Manuel de Vega

**Affiliations:** 1 Departamento de Psicología Básica I, Universidad Nacional de Educación a Distancia (UNED), 28040 Madrid, Spain; 2 Instituto Universitario de Neurociencia (IUNE), Universidad de La Laguna, Tenerife, Spain; 3 School of Foreign Languages, Dalian Maritime University, Dalian, China

**Keywords:** Negation processing, Inhibitory mechanisms, Neural reuse, Cognitive control, Embodied effects

## Abstract

Negation is known to have inhibitory consequences for the information under its scope. However, how it produces such effects remains poorly understood. Recently, it has been proposed that negation processing might be implemented at the neural level by the recruitment of inhibitory and cognitive control mechanisms. On this line, this manuscript offers the hypothesis that negation reuses general-domain mechanisms that subserve inhibition in other non-linguistic cognitive functions. The first two sections describe the inhibitory effects of negation on conceptual representations and its *embodied effects*, as well as the theoretical foundations for the reuse hypothesis. The next section describes the neurophysiological evidence that linguistic negation interacts with response inhibition, along with the suggestion that both functions share inhibitory mechanisms. Finally, the manuscript concludes that the functional relation between negation and inhibition observed at the mechanistic level could be easily integrated with predominant cognitive models of negation processing.

## Introduction

Negation markers such as *no* figure among the first words uttered by children (e.g., Dale & Fenson, 1996), rank high among the most frequent words in many languages (for example, position 11 in the largest Spanish Corpus, Web-Dialects, 2016), and are used for a wide range of communicative purposes (e.g., rejection, denial, and talk of absence) (e.g., Horn, 1989). And yet, according to the psycholinguistic research, their understanding is often demanding and difficult. In many experimental tasks, sentences that include a negation marker (i.e., negative sentences) take longer to process and produce higher error rate than their affirmative counterparts (for recent overviews: Kaup & Dudschig, 2020; Tian & Breheny, 2019). This so-called negation difficulty is usually explained in one of two possible ways, which are not necessarily incompatible. Negative statements are pragmatically special, as they are more context dependent than their affirmative counterparts, and hence uttered in a rather limited set of communicative situations (e.g., Givon, 1979; Glenberg et al., 1999; Beltrán et al., 2008). Thus, one explanation is that negation might be hard because the conditions surrounding everyday utterances are generally ignored in the controlled setting of laboratory (e.g., Wason, 1965; Giora, 2006; Tian & Breheny, 2019). However, negation is also special from a purely cognitive point of view as it requires, by default, representations and processes which are not regularly recruited for the processing of affirmative sentences, and hence it is in comparison more demanding than the latter (e.g., Carpenter & Just, 1975; Clark & Chase, 1972; Kaup et al., 2007a, 2007b). So, an alternative explanation is that negation difficulty comes from its cognitive complexity, which is easier to assess experimentally. The two-step simulation theory is likely to be the most representative and overarching account of this type (e.g., Kaup & Dudschig, 2020; Kaup et al., 2006). For this theory, negation processing involves a transition between two mental simulations. An affirmative sentence such as *The flower is yellow* calls for only one mental simulation, which directly corresponds to the factual situation being described (a yellow flower). In contrast, the inclusion of a negative marker in the same sentence (*The flower is not yellow*) calls for representing both the negated situation (a yellow flower) and what might be the factual situation (e.g., a flower of a different color). Thus, according to this theory, negation is representationally more complex because it requires two instead of only one mental representation.

To arbitrate between the above two general explanations, most cognitive research on negation has mainly focused on figuring out how it gets represented during comprehension. In contrast, little attention has been paid to other crucial elements for a complete cognitive model of negation: the processes and mechanisms that guide and implement these representations. Certainly, the two-step simulation theory does assume that the transition between simulations implies processes that guarantee the suppression of the negated situation, as well as its replacement by the factual one. Yet, the specific details of these processes have generally not been provided. Notably, this situation has changed in the last years. Several groups have started to independently emphasize the relevance of general-domain cognitive control mechanisms for negation processing, connecting in this way the study of negation with broader conceptualizations in psychology and neuroscience of language (e.g., de Vega et al., 2016; Dudschig & Kaup, 2018; Beltrán et al., 2019; Wirth et al., 2019). In particular, these proposals are akin to views of language processing that relies primarily on non-linguistic mechanisms (e.g., Glenberg & Gallese, 2012; Hasson et al., 2018), and more specifically, to the idea that cognitive control could be responsible for managing competing representations in both language production and comprehension (e.g., Novick et al., 2010; Nozari & Novick, 2017). In the case of negation, cognitive control is supposed to monitor the competition between representations, and to help to resolve it in one specific direction: deactivating the representation of the negated information, and giving prominence to the factual situation (e.g., Beltrán et al., 2019; Dudschig & Kaup, 2020a, b). In this manuscript, the focus will be on the mechanisms that implement the deactivation or inhibition of the negated situation.

The manuscript presents the hypothesis that negation processing relies upon the reusing of general-domain inhibitory mechanisms. Section 2 describes the literature supporting that negation has inhibitory consequences for the information under its scope. Next, Sect. 3 exposes several theoretical arguments that aim to connect negation with the mechanisms of behavioral and cognitive control, and particularly, with response inhibition. Section 4 presents empirical evidence showing that linguistic negation interacts with response inhibition, along with the suggestion that both functions share inhibitory mechanisms. Finally, Sect. 5 concludes by providing suggestions for integrating the neural reuse hypothesis into other conceptualizations of negation processing (specially, the two-step theory).

## Negation has Inhibitory Consequences

There are two lines of research that independently supports that negation has inhibition-like consequences. The first line of research is composed of studies, using the so-called *conceptual probe paradigm,* that assessed the level of activation and recall of negated concepts relative to non-negated ones. The second one includes studies on the modulatory effect of negation on the so-called *embodied effects*, namely, on how negation modulates the differences in sensorimotor network involvement between action and non-action language.

In the *conceptual probe paradigm,* the activation level of a critical concept is inferred from its availability in working memory, indexed by the ease of processing it in a later task (Mckoon & Ratcliff, 1980). The basic trial procedure consists of a sentence containing a critical concept, followed by a probe stimulus (word or image) that corresponds or not to the critical concept. The relevant measurements are the latency and accuracy associated with the response (e.g., naming, lexical decision or recognition) to the probe stimulus. Shorter latency and/or lower error rate are supposed to indicate higher availability of the concept in working memory. When employed to examine negation processing, this paradigm has revealed longer latency and higher error rate for negated than non-negated concepts, suggesting thereby that negation reduces availability in working memory and hence, suppresses the information under its scope. To illustrate, consider MacDonald and Just (1989), who possibly reported the first study to compare negated and non-negated concepts with the probe paradigm. These authors presented participants with sentences like “Every weekend, Maria bakes some bread but no cookies for the children”, and immediately afterwards measured the activation level of the relevant concepts (“bread”, “cookies”), by asking for either recognition (Experiment 1) or naming (Experiment 2) of the probe stimulus. The results showed longer reaction times for probes related to the negated words than those related to the non-negated words (“cookies” vs. “bread”, respectively), irrespective of the position of negation in the sentence. Further research found similar results, using variations of the basic probe paradigm, confirming thereby that negation somehow suppresses the information (or concept) under its scope (e.g., Kaup & Zwaan, 2003; Kaup et al., 2006; Mayo et al., 2004). Note that this interpretation of the results with the probe paradigm is only justified if the negated concept has been represented similarly to non-negated ones during the early stages of sentence comprehension. Without such initial activation of the negated information, it would be unwarranted to label these effects as suppression, instead they would be reflecting “no-activation”, or as much “proactive inhibition” of the concepts under negation scope. Nonetheless, there is a significant body of evidence indicating that negated and non-negated concepts are equally available in working memory at the initial stage of sentence processing, which supports that suppression underlies the negation effects on conceptual activation (e.g., Hasson, & Glucksberg, 2006; Kaup et al., 2006, 2007a, 2007b).

Diminished availability in working memory has consequences that could go beyond immediate recognition to affect subsequent recalling. A well-known principle in psychology is that the worse a stimulus is encoded, the poorer the memory of it would be (e.g., Tulving, & Thomson, 1973). So, negation-induced suppression might be expected to impact also the long-term memorability of the negated concepts (Cornish & Wason, 1970; Howard, 1975; Mayo et al., 2014). Mayo et al. (2014) recently gave the most systematic demonstration of this effect. In four experiments, these authors showed participants videos and verbal stories describing everyday places and situations (e.g., a video of a virtual walk through an apartment). Immediately after the presentation of these stimuli, they asked participants about the properties of some of the entities shown (e.g., about the type of wine). For some questions, the correct answer was “yes” (e.g., was it white wine?), because the property described in the question matched with the actual property of the shown entity. For the others, the correct answer was “no” (e.g., was it red wine?), due to the mismatch between the shown entity and the description in the question. In a final experimental phase, they unexpectedly asked participants to freely recall as much information as they could from the video or the text (e.g., wine). The results showed that the number of entities recalled was smaller when the response in the verification phase was “no” than when the response was “yes”. Accordingly, they concluded that actively negating the property of an entity (e.g., wine color) leads to the forgetting of the entity itself (e.g., wine). Recently, Zang et al. (in preparation) have replicated and extended these results, and confirmed accordingly that negation not only reduces the immediate availability of concepts but also their memorability. Therefore, both the probe paradigm and the memory experiments suggest that negation has inhibitory effects. Nonetheless, in the psycholinguistic literature, there are other studies that have failed to obtain evidence for negation-induced concept suppression (for a review, Tian & Breheny, 2019). Discourse demands, pragmatic knowledge and context information have been found to change the two-step sequence for negation processing, by bolstering instead of suppressing the negated concept in some cases (e.g., Autry, & Levine, 2012; Giora et al., 2007), or by skipping the initial activation (and representation) of the information under its scope in others (e.g., Dale & Duran, 2011; Tian et al., 2010; Orenes et al., 2014; Orenes et al., 2016). Thus, negation seems not to suppress concepts in all instances.

Research conducted within the embodiment approach to meaning is equally consistent with the idea that negation induces inhibitory effects. On the premise that linguistic meaning is grounded in the activity of sensorimotor brain networks, several groups of researchers have assessed how negation modulates well-known *embodied effects*. The treatment we offer here on this literature will necessarily be brief (for a more comprehensive coverage, Papeo & de Vega, 2020). Our aim is simply to highlight how, despite of the variety of paradigm and measurement techniques used to obtain *embodied effects*, the very same upshot emerges for negation. More specifically, this literature reveals that negation modulates *embodied effects* by reducing the differences between action and non-action language in: (i) the BOLD signal from premotor and motor cortical areas (Tettamanti et al., 2008; Tomasino et al., 2010), (ii) the corticospinal reactivity induced by stimulation of primary motor cortex with TMS (Liuzza et al., 2011; Papeo et al., 2016), (iii) the reaction times and error rates for motor actions that are performed in parallel with the processing of linguistic material (Aravena et al., 2012; Bartoli et al., 2013; García-Marco et al., 2019), and (iv) the power desynchronization of Mu sensorimotor rhythms (Alemanno et al., 2012). For all of these measures, the main finding is that, for affirmative sentences, action language shows greater activity than non-action language, while negation blocks these differences, often by directly reducing the sensorimotor network activation associated with action language processing. This means negation somehow inhibits neural activity in meaning-related networks; a conclusion that has led some authors to go further and propose that negation meaning is grounded on such “disembodied effects” (Bartoli et al., 2013; Pulvermüller, 2018). Regardless of the theoretical interpretation of the finding, the critical point here is that the embodiment literature also supports the view of negation as inducing inhibitory effects.

Although, overall, they converge on revealing the importance of inhibition for negation, the results from the probe paradigm and the embodiment research diverge as to the chronometry of the inhibitory effect on negated information. Taking advantage of the basic structure of the probe paradigm, several studies manipulated the time interval between the sentence offset and the probe onset, with the goal of examining the temporal dynamics of negation processing (e.g., Hasson & Glucksberg, 2006; Kaup et al., 2006). In these studies, the suppression effect was found for longer intervals between the sentence and the probe (approximately 1 s), while in the case of shorter intervals, negated and non-negated concepts showed similar response times. This pattern of results is consistent with the predictions of the two-step simulation theory and suggests that negation processing is non-incremental, as inhibitory consequences happen only after the initial activation of the negated concepts. However, this conclusion contrasts with the dynamic revealed by some of the results from the embodied literature. For example, in a TMS study, Papeo et al. (2016) measured corticospinal reactivity while participants read affirmative and negative action sentences ("Now I write" and "I do not write"), using three different intervals between verb onset and the TMS pulse (250, 450, and 550 ms). Already at the shortest interval (250 ms), negation reduced the difference between action and non-action verbs, suggesting inhibition activity from early stages of meaning processing. Similarly, Liuzza et al. (2011) identified negation effect on corticospinal reactivity at 500–800 ms after the onset of sentences such as “I/do not squeeze lemon”, and Aravena et al. (2012) observed that negation attenuated grip strength at 300 ms after the onset of verbs describing hand actions. Thus, unlike concept suppression, the inhibitory impact of negation on sensorimotor brain networks is apparently fast, suggesting that the processing of negation might be incremental.

This discrepancy between the two lines of research might be reconciled if recognizing their differences in methodology and purpose. A particularly relevant difference concerns the type of data used to infer inhibition-like effects. The *probe paradigm* involves an explicit judgment or action on the probe stimulus (recognition, naming, lexical decision), which is performed after sentence completion and presumably reflects the activation level of the concept within the simulation of the whole sentence. In contrast, cortico-spinal reactivity and motor action interference are indirect, and to some extent automatic measures, which do not require explicit judgments on the negated information, possibly indexing early meaning processing. Therefore, embodied effects are not snapshots of the working memory status for the whole sentence representation, but reflect the low-level mechanisms that serve the integration of lexical meaning (e.g., Pulvermüller, 2018). This possibility suggests that both literatures are surely reflecting different aspects of the temporal dynamic of negation processing and could hence be integrated somehow. Nonetheless, how to integrate them in a common theoretical framework is a question that requires further research.

## Negation Could Reuse Inhibitory Mechanisms

The results described in the preceding section support the intervention of inhibitory mechanisms in the processing of negation. Yet, they fall short of specifying these mechanisms, revealing only the consequences of negation on the information under its scope. In this section we present some theoretical arguments that point to the possibility that negation operates by recruiting domain-general inhibitory mechanisms that are critical for behavioral and cognitive control.

One argument concerns the ontogenetic trajectory for the acquisition of negative verbal expressions. Some specific features of this trajectory are still under debate (e.g., Austin et al., 2014; Feiman et al., 2017; Nordmeyer & Frank, 2014). However, there is one uncontroversial fact: the earliest negation utterances in children are mostly produced to communicate *rejection* and *prohibition* (Bloom, 1968; Pea, 1982). Expressions such as “no go outside” are frequent between 13 and 16 months of age, and precede other negations that fulfill more abstract communicative functions, such as *denial* (e.g., “no, apple” to answer the question “is that a biscuit?”). Children and adults produce such negative expressions to reject an object, or to somehow stop or prevent an imminent action, establishing thereby a strong association between the verbal markers of negation and the rejection and prevention of an action. Moreover, in this early stage of linguistic development, children often talk to themselves using negative expressions as self-prohibition commands, showing that they have already internalized negation as an important tool for self-regulation (e.g., Choi, 1988; Pea, 1982). Thus, in the one-word developmental stage, negations are frequently used in contexts that imply a close association with behavioral control, and in particular, with the stopping and prevention of actions. Of note, the verbal expressions of rejection and prohibition go hand in hand with the production of head-shaking gestures for the same communicative purposes, which precede the acquisition of nodding gestures used to communicate acceptance (e.g., Kettner & Carpendale, 2013). Regulating behavior provides then the context for first symbolic expressions of negation.

The ontogenetic trajectory of negation has led to claim that its more abstract expressions (e.g., *denial*) derive from previously acquired functions, with the formers being just logically and metacognitively sophisticated versions of rejection (Russell, 1948; Wason & Johnson-Laird, 1972). In line with this speculation, *denial*, the most frequent pragmatic function of negation in adults, has been seen as implying rejection (of a proposition), with disagreement expressions as ontogenetically bridging between them (Hummer et al., 1993; Dimroth, 2010). Thus, there seems to be unicity across the diverse pragmatic functions of negation. The question is: what could be the source for this unicity? From a cognitive point of view, the response is that they are connected because they all rely upon the same default computation, which is realized by a similar set of cognitive processes. Most cognitive models have assumed one way or another that negation is one specific type of computation, aiming to somehow reverse (we would say, reject) the meaning or the truth value of the negated information (e.g., Carpenter & Just, 1975; Mayo et al., 2004; Kaup et al., 2007a, 2007b). In fact, the two-step simulation theory claims that the transition between the two simulations is the default way to process negation, and assumes accordingly a cognitively-based unicity. In this theory, rejection plays a critical role, manifested in the suppression of negated information; however, as noted above, negation-induced suppression has not been well characterized at a mechanistic level. One possibility is that, given its early association with the rejection and prevention of actions, negation somehow recruits the mechanisms subserving the more general purpose of inhibiting actions and thoughts. This proposal fits well into a current theoretical framework that views recycling and reusing as fundamental principles of brain organization and function.

The idea that the brain recycles the available mechanisms for new functional traits instead of creating *ex novo* ones has a long tradition in neuroscience and evolutionary biology (e.g., Dehaene & Cohen, 2007; Fitch & Martins, 2014; Gould & Vrba, 1982; Lashley, 1951). Recently, Anderson (2010) has pushed it forward by proposing a set of general principles of brain functional organization, which are known as the neural reuse theory. The basic premise, shared with prior conceptualizations (e.g., Lashley, 1951; Gould & Vrba, 1982), is that newly acquired cognitive functions reuse preexisting brain mechanisms which subserve phylogenetically more ancient functions. Crucially, the reuse is not assumed to happen indiscriminately but depends on the functional equivalence between the new and preexisting functions, such that the more the similarity between them, the more likely the preexisting mechanisms will be co-opted for the new purpose (Anderson, 2010). Moreover, the neural reuse theory predicts mutual influences between functions, manifested as either facilitation or interference effects. Overall, the proposal that negation reuses inhibitory mechanisms is an outstanding example of the neural reuse hypothesis, which in addition meets the functional equivalence principle. Under close examination, there is a strong functional resemblance between negation processing and response inhibition, as implemented in the *Go/NoGo* and the *Stop Signal* tasks (e.g., Huster et al., 2013; Verbruggen & Logan, 2008, 2009).

The creation of a strong tendency to respond is the critical process in response inhibition tasks. For the Go/NoGo task, this tendency is settled by presenting, in fast succession, a larger number of response (Go, 70–80%) than inhibition (NoGo, 30–20%) trials. In the *Stop Signal* Task (SST), the tendency is generated directly on each trial, wherein a cue is first displayed instructing the participant to select one out of the two possible responses, followed in some trials by a signal presented with a short and variable delay requiring them to stop the selected response. Thus, similar to negation-induced suppression, response inhibition requires firstly the activation of a response program which would later be stopped or suppressed if inhibition is to be successful. To some extent, this means that both can be considered functionally equivalent, but falls short of demonstrating that negation shares inhibitory mechanisms with response inhibition. Empirical evidence in two directions is needed: first, directly demonstrating that negation and inhibition activate similar brain networks independently; second, finding mutual influences between them when they are executed simultaneously, which could be manifested in behavioral facilitation or interference, or interactions at the neurophysiological level.

In sum, both the ontogenetic trajectory and the neural reuse theory are arguments in favor of the hypothesis that negation reuses the basic and domain-general inhibitory mechanisms frequently associated with response inhibition. More broadly, this conclusion is also consistent with other phylogenetic conceptualizations of language origin, especially those arguing that language co-opted and co-evolved with already existing complex motor functions, which were there to support planning in the face of lithic technology (Stout & Chaminade, 2012) or cooperative communication through gestures (Arbib, 2012). The next section presents recent neurophysiological research that examined the mutual influences between negation and response inhibition, with the aim of providing empirical evidence for the reuse hypothesis.

## Negation Interacts with Response Inhibition

The first EEG study assessing the interaction between negation and response inhibition was conducted by de Vega et al. (2016). These authors implemented a dual-task paradigm that has served as reference for subsequent studies, in which the comprehension of affirmative and negative sentences was combined with the performance of a Go/NoGo task. Participants read word by word imperative action sentences such as “Now you will [will not] cut the bread”, and a visual cue (circle) would appear above the action verb shortly after its onset (300 ms). 70% of the trials received a Go cue, indicating that participants should make a response by pressing a certain key on the gamepad; while the remaining 30% of the trials received a NoGo cue, for which participants were required to withhold their response. Regarding the EEG analysis, they focused on measuring the influence of sentence polarity on typical electrophysiological signatures of response inhibition, in particular the power in theta frequency range in fronto-central sites, which is generally much stronger for NoGo (inhibition) than Go (response) trials (Huster et al., 2013). The change in theta power confirmed the expected inhibitory effect, validating it as a marker of inhibitory processes. More importantly, it was modulated by sentence polarity. For NoGo trials, negative sentences diminished fronto-central theta power compared to affirmative sentences, whereas there was no effect of polarity for Go trials. Such interaction between cue and polarity supported the idea that negation processing shares neural mechanisms with response inhibition. Subsequent research has addressed some of the issues raised from this original study.

A key issue concerns the generalizability of the modulatory effects of negation on response inhibition. The finding in de Vega et al. (2016) concerned negated actions in imperative form and a very specific inhibition task, the Go/NoGo. Yet, the neural reuse hypothesis implies a much broader scope, as it predicts that negation makes use of general-domain mechanisms regularly, and not only to express the prevention of an action. Thus, the interaction found by de Vega et al. (2016) provided only a first but still limited proof of the general hypothesis. In the following years, several studies analyzed whether in fact the interaction effect extends to other situations: non-Indo-European languages with different writing systems and neural demands, linguistic materials belonging to semantic domains other than action language, and other inhibitory tasks besides the Go/NoGo paradigm. Adopting a similar dual-task paradigm as in de Vega et al. (2016) and Liu et al., (2020a, 2020b) tested whether comprehension of negative imperative sentences in Mandarin modulates subsequent inhibitory processes involved in the Go/NoGo task. Negation was found to be associated with reduced amplitudes of ERP N2 compared to affirmation in NoGo condition, but they did not differ in Go condition. Along with the P3, the N2 component forms a well-known fronto-central ERP signature of the inhibitory mechanisms operating in response inhibition tasks (e.g., Huster et al., 2013), and accordingly, the results in this study support the cross-linguistic generality of the negation-to-inhibition modulation.

Inhibitory consequences of negation have been observed using different linguistic materials (e.g., Kaup & Dudschig, 2020). If the recruitment of inhibitory mechanisms is a general feature of negation processing, then similar interactions between negation and inhibition could be expected for semantic domains and sentence structures other than action-related imperatives. In a follow-up study, Beltrán et al. (2019) employed the same dual-task paradigm as de Vega et al. (2016), with the novelty of including imperative sentences referring to both physical actions and mental events (e.g., “Now you will [will not] wish a surprise”). The results showed that, irrespective of the semantic domain, negation reduced the power of the fronto-central theta rhythm associated with NoGo trials, while there was no difference between affirmative and negative sentences for Go trials. Furthermore, they also found an identical interactive pattern for the beta frequency range in right-frontal sites. Importantly, this latter finding is consistent with recent research establishing that increased beta power in right-frontal brain areas is a critical signature of the inhibition of motor actions (Castiglione et al., 2019; Swann et al., 2009; Wagner et al., 2018). Thus, the sharing of inhibitory resources between negation and response inhibition is not limited to action language, but extends to other semantic domains as well.

However, does the negation-to-inhibition modulation exist only in negative imperative sentences that express prohibition? The performance in the response inhibition task is likely guided by mental representations that link the cues to a specific demand to perform an action in Go trials (e.g., “respond if the cue is yellow”) or to prohibit the action in NoGo trials (e.g., “do not respond if the cue is blue”). Therefore, the interaction between negation and response inhibition in prior studies might well be caused by this close similarity in functions and meanings between the negative imperative sentences of the comprehension task and the mental representations of the NoGo cue for the inhibition task. To rule out this alternative account, Liu et al. (in preparation) employed a dual-task Go/NoGo paradigm with existential instead of imperative sentences as stimuli, for example, “There are already/no apples in the fridge”. For these sentences, the Go/NoGo cues appeared 300 ms after the presentation of the object noun (e.g., apples). The results showed again an interaction between Go/NoGo cue and sentence polarity. In this case, the interaction arose in the P3 component of the fronto-central N2-P3 ERP complex, which shows generally larger amplitudes for NoGo than Go trials (Smith et al., 2008). In Liu et al.’s study, the P3 amplitude was larger for negative than affirmative sentences in NoGo conditions, whereas there was no polarity effect for Go trials. This finding indicates that the interaction with response inhibition extends beyond imperative prohibitions to other types of negative sentences, particularly those expressing absence/non-existence. Even more importantly, it supports the still speculative possibility that the same type of inhibitory mechanisms is involved in the processing of most functional types of negation.

The generalizability of the reuse hypothesis requires as well interactions of negation with other motor and cognitive inhibition tasks. To date, no study has examined it for cognitive inhibition. However, Beltrán et al. (2018) recently investigated it with a different motor inhibition paradigm: the SST. As in previous studies, the critical time to assess polarity influence on electrophysiological signatures of inhibition was the processing of verb in imperative sentences (e.g., “Now you will [will not] cut the bread”). Shortly after the verb onset, an arrow (Go cue) pointing to the left or to the right appeared on the screen, indicating that participants should press a gamepad key using the corresponding left or right hand. Critically, in half of the trials, they also received an auditory Stop cue, at a variable interval from the arrow onset, indicating that they should withhold the selected response. As standard for the SST, this variable interval between the Go (arrow) and Stop (auditory signal) cues (Stop Signal Delay, SSD) was critical to generate equivalent distributions of successful (inhibition) and unsuccessful (non-inhibition) Stop trials. This time the interaction between cue and polarity was manifested in an early EEG signature of inhibition, the N1 ERP component, which typically shows larger amplitudes for successful Stop (inhibition) than both unsuccessful Stop and Go trials, and is thought to be an equivalent in the auditory domain of the inhibition-related fronto-central N2 component (Kenemans, 2015). The interaction was due to increased N1 amplitudes for successful stops in the context of negative sentences compared to affirmative ones, while there were no sentence polarity effects for either unsuccessful Stop or Go trials. In addition, this study generated other two important results. First, brain sources analyses identified the right inferior frontal gyrus as the likely generator of the effects in the N1 component, a key region in the inhibition neural network (e.g., Aron et al., 2014). Second, at the behavioral level, the Stop Signal reaction time (SSRT), an estimate of the time required for inhibition, was longer for negative than affirmative sentences. The latter result has been very recently confirmed by a different group of researchers (Montalti et al., 2021). Thus, the interaction between negation and response inhibition is not restricted to one particular response inhibition task, as it is obtained for both the Go/NoGo and Stop Signal tasks. Whether or not it generalizes to cognitive inhibition tasks (e.g., suppression of memory retrieval, Anderson & Hanslmayr, 2014) is still a matter for further research.

The evidence described so far reveals interactions in which sentence polarity modulates signatures of response inhibition. However, a modulation in the reverse direction should be expected if, as predicted by the reuse hypothesis, negation and inhibition shares inhibitory resources. Liu et al. (2020) tested this prediction by adapting the original Go/NoGo dual task paradigm. The main change consisted in the relocation of the Go/NoGo cues within the trial. So, instead of appearing shortly after the verb onset, the cues were presented 1200 ms before the onset of the first sentential word. This allowed to analyze the influence of the Go and NoGo cues on the ERP waveforms elicited by the word-by-word sentence comprehension. As predicted, they found an interaction between Go/NoGo cue and sentence polarity. Again, there was no difference between affirmative and negative sentences after the Go cue (response). In contrast, after the NoGo cue (inhibition), there was a sustained ERP amplitude difference between affirmative and negative sentences, which started just after the polarity marker onset. Negative sentences following NoGo cues showed more negative waveforms than all the other conditions. In addition, brain sources analysis identified two inhibition-related brain regions as the likely origin of this interaction, the right inferior frontal gyrus (rIFG) and left middle frontal gyrus (lMFG). In both sources, negative sentences after NoGo cues showed stronger activations than all the other conditions. The sustained ERP effect of this study cannot be directly interpreted as a neural signature of inhibitory mechanisms. The is reason is that, unlike the fronto-central theta power and N2-P3 ERP complex, it does not result from comparing inhibitory with non-inhibitory trials in the context of a response inhibition task. Nonetheless, this ERP effect reveals an interaction in the predicted direction, which encourages the idea that linguistic negation reuses inhibitory processes; an interpretation that is strengthened by the source-localization of the interaction at regions that are core components of the inhibitory neural network (Aron et al., 2014). Thus, there seems to be bi-directional modulations between negation and response inhibition, a finding that is generally interpreted as supporting conclusions of resource sharing between two cognitive functions (e.g., Pessoa et al., 2012; Agudelo-Orjuela et al., 2020).

For all the above EEG research, the same general type of interaction between negation and response inhibition arose over and over again at well-known neurophysiological markers of inhibitory mechanisms. When the influence of negation on response inhibition was examined, the interaction reflected an effect of polarity on inhibitory trials, but not on trials involving the delivering of a motor response. Similarly, when the tested influence was of response inhibition on negation processing, the upshot was a modulation by the processing of negative sentences but not affirmative ones. Overall, this interaction pattern is completely consistent with the neural reuse hypothesis. Firstly, it reveals that the modulatory effect of negation only happens for trials demanding inhibitory mechanisms (i.e., NoGo or Stop trials); in contrast, negation has no effect when these mechanisms remain in a dormant state (i.e., Go trials). Secondly, it indicates that the processing of negative sentences is affected by prior activations of inhibitory mechanisms, but not by the prior delivering of a response. Thus, it shows that negation and response inhibition modulate each other in a rather specific way. Finally, it is worth noting that the reuse hypothesis could have been readily disproved if other results had emerged in the above research. For example, main effects of either polarity or response inhibition, without any interaction between them, had spoken against a sharing of neural resources. And similar doubts had arisen as well from interactions driven by either an effect of polarity on response trials, instead of an effect on inhibition trials, or an effect of trial type (either response or inhibition) on affirmative sentences, but not on negatives.

Although negation modulated the SSRT in the SST, all the studies using the Go/NoGo task failed to find behavioral evidence of negation-to-inhibition influence. The lack of modulation in these studies is likely due to the fact that both response execution (Go) and inhibition (NoGo) showed a virtual ceiling effect. In the standard Go/NoGo task, a short inter-trial interval is normally implemented to increase the errors rate (Zamorano et al, 2014). However, in the above dual-task paradigms, the inter-trial interval was much longer than in the standard version, because of the time required to read and comprehend the sentence that was presented word by word. Surely, this hindered the formation of the typical strong tendency to respond, leading to the noted virtual ceiling effect and the failure in finding polarity modulation on Go and NoGo errors. Nonetheless, the results for the SSRT in Beltrán et al. (2018) and Montalti et al. (2021) indicate that negation modulates behavioral signatures of response inhibition. Unlike the Go/NoGo task, the SST induces a stronger tendency to respond in every trial, reducing so the dependency on both short inter-trial intervals and unbalanced probabilities between execution and inhibition trials. This suggests that the SST might be a better candidate for testing the interaction between negation and response inhibition by placing strong competition for inhibitory resources. A very recent finding has added further support to the functional importance of the reuse of domain-general inhibitory resources by negation (Dudschig et al., 2021), revealing that reaction time differences between affirmative and negative instructions (i.e., the negation effect) were larger in children with ADHD, who often show difficulty in inhibitory control tasks (e.g., the SST), than in a control group.

In sum, there is growing evidence that negation modulates electrophysiological and behavioral signatures of response inhibition. This happens for both alphabetic Indo-European languages and logographic Mandarin, for linguistic materials that differ in both semantic domain and negation type, and for experimental paradigms involving different response inhibition tasks. In addition, the interaction between negation and inhibition also manifests in the reverse order, that is, from response inhibition toward negative sentence processing. Together, these results are consistent with the hypothesis that negation shares domain-general inhibitory mechanisms with other cognitive functions, and going a step further, that it might reuse these mechanisms when producing its often-reported inhibitory consequences.

## Revisiting the Two-Step Dynamic

The finding of interactions between negation and response inhibition is, at first, consistent with the two-step theory of negation. Negated information is first mentally simulated and afterward suppressed by recruiting domain-general inhibitory mechanisms. However, as for the findings concerning negation impacts on *embodied effects*, the timing of the interaction is not apparently consistent with the sequential and non-incremental dynamic proposed by this theory. In all the studies reviewed in the previous section, the interaction happened just after the polarity marker onset, during sentence verb processing, suggesting that the inhibitory mechanisms were somehow recruited before the representation of the whole sentence meaning. As noted in Sect. 2, this discrepancy might be only reflecting the complementarity between the results that supported the development of the two-step simulation theory and those obtained by research conducted at a more mechanistic level. Therefore, one reconciling possibility is that, as soon as the negative operator is read (or heard), it recruits the relevant inhibitory neural mechanisms and starts constraining neural meaning processing for the upcoming sentence terms. This constraint might be strong enough as to modulate lexical processing in meaning-related areas, by reducing for example activity in sensorimotor network for action language, but insufficient to block the simulation of negated information. In this way, the interaction with response inhibition and the attenuation of *embodied effects* complement the two-step dynamic, by indicating that negation is not completely neglected until a second sentence simulation stage; instead, it might activate some critical mechanisms since the very onset of the marker.

There is at least one potential problem with this integrative picture. Most ERP studies found no evidence of the constraints imposed by negation on lexical processing, indicating seemingly a “clean” simulation step for the negated information. More specifically, negation seemed not to reverse (or reduce) the semantic relatedness effect that is typically obtained for the N400 component (e.g., Fischler et al., 1983; Kounios & Holcomb, 1992; Dudschig et al., 2019; Palaz et al., 2020). In fact, both affirmative and negative sentences showed the same type of relatedness effect, namely: larger N400 amplitudes for semantically unrelated than related words. However, within the ERP literature, polarity has occasionally produced differences which either overlap in time with the N400 semantic-relatedness effect (e.g., Xiang et al., 2016; Sommer et al., 2021) or arise immediately afterwards (e.g., Lüdtke et al., 2008; Farshchi et al., 2020), suggesting that negation effects happen earlier than predicted by a strict two-step simulation dynamic. Negation might not reverse yet the sentence meaning in these early stages but somehow it changes the processing of the upcoming lexical terms. Indeed, one might argue that, given the strength of the semantic-relatedness effect in N400, the focus on its reversion (or reduction) has likely hindered the finding of more subtle negation influences. Therefore, the abundant ERP literature does not in principle oppose to an early recruitment of inhibitory mechanisms that operate in parallel with the simulation of negated information.

Yet, conceptually, one intriguing question is why to initiate mechanisms that have to wait until the negated information is represented. Wouldn’t it be better to start recruiting these mechanisms when suppression is going to operate fully? The neural reuse hypothesis assumes that the inhibitory mechanisms have been strongly associated with symbolic markers of negation by experience. Accordingly, it predicts that the onset of any negation marker triggers immediately and automatically these mechanisms (e.g., Pulvermüller, 2018). The results described in Sect. 4 are, overall, confirmations of this prediction. The problem is that, according to the proposed integrative hypothesis, these mechanisms remain to some degree on standby until the negated information is fully processed. The examination of pragmatic factors might help to clarify why this happens after all.

Most studies on negation processing, including those about *embodied effects* and the reuse of response inhibition, have employed stand-alone, non-pragmatically licensed sentences. Yet, negation is rarely expressed in this way in everyday conversations. When rejecting or prohibiting actions, or when disagreeing or denying propositions, the to-be-negated information is somehow known before negation is expressed (e.g., Horn, 1989). This means that, in everyday conversation, the inhibitory mechanisms might operate immediately on to-be-suppressed information that is already there. In line with this possibility, there is evidence that pragmatically licensed negations are more easily processed, because overall they create strong expectations for the negated information (e.g., Nieuwland & Kuperberg, 2008; Orenes et al., 2016; Schiller et al., 2017; Tian et al., 2010). In contrast, there is no such anticipation for unlicensed sentences, and consequently, although negation markers immediately recruit inhibitory mechanisms, they only operate fully after the negated information is read and represented. Accordingly, this integrative proposal predicts modulations of the inhibitory mechanisms as a function of the expectations created for negated information, allowing then to integrate the important literature on the facilitatory role of pragmatic factors in negation processing.

The focus of this manuscript has been on the inhibitory mechanisms underlying negation, however, the neural reuse hypothesis has a broader scope, covering as well processes and mechanisms that are equally relevant to behavioral and cognitive control. As noted, the earliest negation utterances happen in contexts of rejection and prohibition, and hence at the service of behavioral regulation. Yet, in these contexts, the stopping or prevention of actions is not the only salient feature, as conflict also plays a crucial role. Conflict is often characterized as the competition between two opposed action courses, implemented by detection and adaptation mechanisms and thought to be critical for response inhibition (Botvinick et al., 2001). Indeed, standard inhibitory tasks capitalize on conflict mechanisms, which somehow trigger inhibition as a way of resolving competition and adapt performance for subsequent trials. Thus, given the connection between conflict and inhibition, it is not surprising that negation might recruits as well conflict-related mechanisms. Several studies have recently provided preliminary evidence that goes in this direction, revealing the involvement of non-linguistic conflict adaptation mechanisms for negation processing (Dudschig & Kaup, 2018, 2020a; Wirth et al., 2019). Together, these pieces of evidence and those reviewed in this manuscript contribute to better characterize negation at the mechanistic level, namely, as an operation that is implemented by recruiting conflict and inhibitory mechanisms. At the basis of this recruitment are the concept of neural reuse and the early association of negation with regulatory contexts in which rejections and prohibitions play a capital function.

## Conclusions

The above sections have presented both theoretical arguments and empirical evidence that support the hypothesis that negation processing is implemented at the mechanistic level by the reuse of general-domain inhibitory mechanisms. In particular, the connection has been made with the well-known response inhibition functions, occurring when a prepotent or ready-to-go action must be stopped. The neurophysiological interactions between negation and response inhibition reported here suggest that both share similar inhibitory mechanisms. The evidence comes from both alphabetic Indo-European languages and logographic Mandarin, from linguistic materials that differ in both semantic domain and negation type, and from experimental paradigms involving different response inhibition tasks. However, as for every new proposal, there is still a number of unresolved issues and further evidence will be needed to consolidate the neural reuse hypothesis. A future direction for research is to go beyond the dual-task paradigm to examine whether negation and inhibition activate similar brain networks independently. One possible framework would be asking the same participants to perform separately the linguistic and the inhibitory tasks, and using latter multidimensional decomposition techniques to isolate EEG signals that were sensitive to both negation processing and response inhibition. Also, at the neural level, it would be relevant to demonstrate that the activity of the brain inhibitory network is causally involved in the processing of negation, and not just “correlates” with it. This might be done by, for example, inactivating key structures of this network (e.g., right IFG) with repetitive TMS before asking participants to complete a linguistic comprehension task, and examining whether, as predicted, the inactivation has functional consequences for the comprehension of negative sentences. Another avenue for further research might be to test the generalizability of the interaction between negation and inhibition, exploring how negation interacts not only with response inhibition tasks but also with cognitive inhibition tasks, or analyzing whether the inhibitory hypothesis of negation can be extended to functional types of negation other than imperative and existential sentences. Reasonable predictions here are that negation will influence the inhibitory processes triggered by more cognitively-oriented inhibition tasks (e.g., direct suppression of memory retrieval, Engen & Anderson, 2018), and that interactions like the above would be also detected for other functional types of negation (e.g., denial). Finally, although we have exposed how the reuse hypothesis might be integrated with current cognitive data and models about negation processing, empirical evidence is needed to unravel how exactly is the temporal relationship between the early effects obtained at the mechanistic level and the two-step simulation dynamic, with a special focus on the modulatory role of pragmatics.
